# Let the sunshine in? The effects of luminance on economic preferences, choice consistency and dominance violations

**DOI:** 10.1371/journal.pone.0181112

**Published:** 2017-08-04

**Authors:** Paul W. Glimcher, Agnieszka Tymula

**Affiliations:** 1 Institute for the Study of Decision Making, New York University, New York, United States of America; 2 School of Economics, University of Sydney, Sydney, NSW, Australia; Universidad de Alicante, ITALY

## Abstract

Weather, in particular the intensity and duration of sunshine (luminance), has been shown to significantly affect financial markets. Yet, because of the complexity of market interactions we do not know how human behavior is affected by luminance in a way that could inform theoretical choice models. In this paper, we use data from a field study using an incentive-compatible, decision task conducted daily over a period of two years and from the US Earth System Research Laboratory luminance sensor to investigate the impact of luminance on risk preferences, ambiguity preferences, choice consistency and dominance violations. We find that luminance levels affect all of these. Age and gender influence the strength of some of these effects.

## Introduction

Biological studies now clearly indicate that exposure to outdoor light-levels which can range across 6 orders of magnitude in intensity causally influence a range of neuroanatomical circuits and a range of behaviors mediated by these circuits. Dedicated luminance sensors in the human retina carry continuous cardinal information about light levels ranging from bright sunlight (300 watts/m^2^) to the intensity of indoor electric lighting (<1 watt/m^2^) directly to the hypothalamus, an evolutionarily ancient structure located at the base of the human brain. There, this information influences the neural circuits that are now known to regulate when we want to sleep, mood, daily and seasonal patterns of when we are hungry or sated, and a host of other circuits known to be related to our preferences [1–5]. Complementary psychological studies have also made it clear that light levels across the intensity found in the natural environment (independent of its covariates) do influence many of our fundamental time-varying traits and properties, just as would be predicted from an analysis of these luminance-related neural circuits. Absolute luminance level, for example, strongly influences food choice [6] and light levels can exert such a strong effect on mood that a sharp reduction in absolute light levels can induce clinical depression in as many as 10 or 20% of the human population [7]. In fact, a highly effective clinical treatment for this class of depression is simply exposure to additional light [8], a fact that strengthens the conclusion that light itself is a causal actor in psychological state. Recent evidence suggests that mood, as measured reliably and repeatedly by psychologists, can strongly influence all kinds of preferences. At an economic level, there is also now some direct evidence that light levels influence human choice. A growing body of literature has shown, for example, that weather and seasons affect economic outcomes in financial markets [9–14].

Table 1 summarizes what we have learned so far in terms of the impact of weather on financial decision-making. Market returns tend to be lower on more cloudy days. Although the effects are persistent, [10] estimate that they are too small to make weather-based strategies profitable even if the costs associated with frequent trades are fairly modest. Nevertheless, it is clear from these widely cited papers that the way investors set prices in the markets is influenced in some way by weather, with most suspecting that it is through its effect on moods and investor’s psychology. In this spirit, [13,14] argue that seasonal changes in investment in government bonds and mutual fund flows must reflect seasonal changes in investors’ preferences. In contrast to the small effect sizes in stock market studies, in at least one experimental study, [15] found that on bad weather days people are much more risk averse. Since by design the study focused on weeks with extremely good and extremely bad weather conditions, it may have exaggerated the size effect of weather. Another paper suggests that risk preferences of people suffering from seasonal affective disorder change more in response to seasons than preferences of non-sufferers [16]. Using a survey methodology, a recent working paper [17] found that in a representative sample of 1,550 Dutch respondents cloudiness affects ambiguity attitudes in the month of January. On average, on cloudy days participants perceived the ambiguous gambles to be closer by 0.12 in probability equivalent terms to the objective 0.5 winning probability.

**Table 1:** Literature summary.

	dependent variable	weather variable(s)	effect	notes
**stock market papers**				
Saunders, *AER*, 1993	*market returns* (daily NYSE/AMEX value-weighted index)	*cloudiness* = -1 if full coverage = 0 if 30–90% coverage = 1 if 0–20% coverage	0.00051% increase	comparable effects for other indexes
Cao and Wei, *J*. *Banking Finance*, 2001	*market returns* (daily US CRSP-VW)	*temperature*	0.0026 fall	
Hirshleifer and Shumway, *J Finance*, 2003	*market returns*	*cloudiness* (C) = -1 if SCK = >7 = 0 if 1<SCK <7 = 1 if SKC<1 SCK = cloud coverage today–cloud coverage this week	0.011 fall in daily stock returns 0.02% decrease in a probability of positive stock return	Authors estimate that "because (weather-based) strategies involve frequent trades, fairly modest costs eliminate the gains"
Kamstra et al, *AER*, 2003	*market returns* (daily NYSE index)	*length of the night -12* (in hours) *cloudiness* *precipitation*	0.026% increase no effect (once length of night accounted for) no effect	analysis for fall and winter only stronger effects for markets further away from the equator
Kamstra et al. *RAPS*, 2014	*government bonds*	*season*	Risky returns are higher (lower) and risk-free returns are lower (higher) in fall/winter (spring/summer)	
Kamstra t al., *JFQA*, 2016	*mutual fund flows*	*month of the year*	Investors prefer safe (risky) funds in autumn (spring)	
**experimental papers**				
Kramer and Weber, *Soc*. *Psychol*,. *Person*. *Sci*, 2012	*risk aversion* allocation to safe versus risky (50–50) asset	*month* December (2008) versus July (2008 and 2009)	SAD sufferers are more risk averse in winter than non-SAD-sufferers	N = 331 Online survey conducted in 7/08, 12/08 and 7/09. Participants assessed whether SAD sufferer or not.
Bassi, et al, *Rev*. *Finan*. *Stud*., 2013	*risk aversion* Arrow Pratt index of relative risk attitude for powerexpo utility	“good weather” *cloud coverage* good weather: less than 50% coverage on the day *precipitation* good weather = below average rainfall	RRA higher on bad days by: 32% for low stakes 67.2% for high stakes 17.8% for low stakes 30.6% for high stakes	N = 208 Participants randomly assigned to twin sessions in weeks with good and bad weather
Baillon et al, *working paper*, 2014	*ambiguity aversion* probability equivalent for an ambiguous gamble with 50% wining probability	*cloudiness* (0 for clear sky to 9 maximum coverage) *precipitation*, *sunshine and temperature*	0.12 increase no effect	N = 1,550 representative Dutch panel, measurements only in January

In any case, it is clear from Table 1 that although weather has a significant effect on investor behavior, the effects vary largely between the studies. For example, some studies find the effect of cloud coverage significant [9,10] and some do not [11]. We suspect that this is due to misspecification of the independent variable. In our paper we chose to focus on luminance instead of cloudiness because of the well-understood effects of luminance on brain function, and an absence of evidence that cloudiness, per se, influences brain function. There are no sensory receptors in our nervous system that are influenced by cloud coverage. In line with this neurobiological observation, another paper [11] found that when the duration of the light period of the day (which has a much bigger effect on aggregate luminance than does cloud coverage) is accounted for, the effect of cloudiness on market returns disappears. Unlike cloud coverage, luminance can also be easily manipulated through adjustments in indoor lighting systems. Although we rush to clarify that this cannot be done using standard indoor lighting and requires special high-intensity lamps that are specifically designed to imitate both the intensity and spectrum of outdoor lighting.

To our knowledge no study has yet established any direct microeconomic-level link between luminance level (absolute or relative) and risk attitudes or other standard measures of individual-level preferences or choice behavior. Perhaps just as intriguing is the fact that the effect of neither weather nor luminance on choice rationality and stochasticity in choice has ever been examined. To better understand the associations between these variables in a structurally defensible manner, we therefore used an established and incentive-compatible experimental task to measure preferences for risk, preferences for ambiguity, inconsistency and propensity to choose dominated options over a period of two years across 2530 visitors to the US National Academy of Sciences Museum in Washington, DC. We then investigated whether daily changes in surface luminance in the geographical area where our study was conducted could account for some of the day-to-day variation in our study participant’s preferences. Because luminance variation has significant hourly, daily and seasonal components, our assessment relied on direct minute-by-minute measures of luminance in the Washington DC area made by the US National Oceanographic and Atmospheric Administration.

### Risk attitude

Based on the previous associations between absolute luminance level, mood and risk attitude, we hypothesized that exposure to more sunlight would lead to less risk taking. It is now well established that lower mood, or *affect*, is associated with increased sexual [18,19] and health risk taking [20]. While less is known about changes in financial risk taking and mood, we know that positive mood states have been associated with more conservative behavior in risky tasks involving financial rewards [21–23]. There is market evidence that even professional traders change their investment strategies (increase short selling) on more cloudy days [12]. Somewhat in contrast to this evidence, [15] in a laboratory study find that on extremely cloudy days people are more risk averse than on good weather days, an effect which they suggest may be driven by mood changes. The literature has not reconciled these seemingly contradictory pieces of evidence. Interestingly, our evidence supports both our risk attitude and luminance hypothesis grounded mostly in the literature on seasonal depression, and replicates the risk attitudes and cloudiness findings of [15]. These seemingly contradicting results become less inconsistent when one realizes that we know from the meteorology literature that cloud coverage alone does not accurately predict light exposure at the earth surface level (e.g. [24]). Indeed, in our dataset CloudCoverage does explain some daily variation in luminance, but only 7% of that variation.

### Ambiguity attitude

[25] was the first one to introduce the distinction between risk attitudes and ambiguity attitudes. Risk attitudes refer to people’s willingness to take known risks. Ambiguity attitudes represent their attitude towards unknown odds. Although outside the lab, risk and ambiguity attitudes are usually not separately observable and are sometimes jointly referred to as “risk attitude” in common language. We know from previous literature that risk and ambiguity attitudes seem to be distinct preferences that are only weakly correlated [26,27].

Positive affect has been shown to lead to more optimistic beliefs [28]. In general, happy people are more likely to recall happy events and it is hypothesized that they thus may overestimate positive probabilities [21]. We therefore hypothesized that more light exposure will lead to more optimistic beliefs which would manifest in microeconomic behavior as an increased tolerance for ambiguity. (We did not explore whether higher luminance levels alter behavior in strategic games, as might also be expected, in way that could influence financial markets.)

### Choice consistency and dominance violations

The evidence on the impact of affect on the quality of decision-making is scarce, mixed, and only indirectly related to our task. In positive affective states people tend to use more flexible cognitive strategies, are more creative, and choose to spend more time and effort on creative activities (for example [29–31]). At the same time positive affective states are generally associated with less data-driven and less thorough decision-making and therefore harm the performance in the types of tasks that rely on these skills [32–34]. Based on this evidence, we hypothesized that in our financial decision-making task, that requires no creativity and flexibility but rather clear trade-offs between risks and rewards, propensity to choose dominated options will increase and consistency will decrease as luminance levels increase.

Overall based on the literature in biology, neuroscience and psychology, we formed three hypotheses that we test in the paper:

*Hypothesis 1: Increased luminance will be associated with less risk taking*.
*Hypothesis 2: Increased luminance will be associated with more ambiguity tolerance*.
*Hypothesis 3: Increased luminance will be associated with greater inconsistency in choice and more dominance violations*.

To test these hypotheses, we collected data daily over a period of two years. This allowed us to construct a much richer dataset (with significant daily and seasonal variation in luminance) than in any previous work using experimental tasks. This allowed us to study the effects of not only relative and extreme but also absolute and small weather changes on behavior in one of the largest experimental datasets of individual behavior under risk and ambiguity. We collected individual demographic and socioeconomic variables on our subjects allowing us to both control for these in our analyses and assess whether the weather effects are mediated through them.

In line with our hypothesis, we found that increased luminance leads to less risk taking. This effect was stronger in older participants. When current luminance was high relative to the luminance in the past two days, people were more ambiguity tolerant. When luminance was high, people violated first-order stochastic dominance more and were more inconsistent in their choice. This effect was particularly strong for men. Overall, the effects are not of an enormous magnitude, but nevertheless they are consistent, significant, and strong enough to be expected to have significant effects on financial markets.

## Materials and methods

The New York University and the National Academy of Sciences’ Institutional Review Boards approved research. Data was collected at the National Academy of Sciences Museum in Washington, DC. Three touch screens were mounted in a kiosk at the museum and were used to collect responses from the study participants as a part of a larger exhibition on aging (Life Lab: Aging). In the paper we present incentive compatible data collected over a two-year period (from May 2012 to May 2014) from these kiosks.

Museum visitors, who were interested in exploring the exhibit, were offered the opportunity to make binary choices, which would provide information about their risk-attitudes. Before beginning to make choices, subjects were asked whether they consented to participate in a research experiment. Independent of their decision, their experience with the exhibit was exactly the same. Data from subjects who did not consent are not included here. Since we could not secure informed consent from children and their guardians in this setting, our sample includes only people 18 years old and older. Non-consenting subjects thus include all minors; no information about the age distribution of non-consenters is available, by design. S1 Fig in the Supporting information explains the procedure that was used to assess whether the museum visitor qualifies to participate in the study.

**Fig 1:**
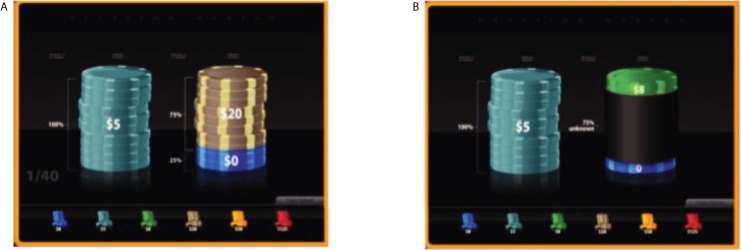
Design. **An example of A. risky and B. ambiguous trial.** A: the participant is choosing between $5 for sure (left) and a 75% chance of winning $20 (right). B: the participant is choosing between $5 for sure (left) and an ambiguous probability of winning $8 (right). The exact odds of winning $8 are somewhere between 25% and 75% (ambiguity level = 50%).

The instructions for the task and the task itself were implemented through a touch screen interface. The task was based on our earlier papers on preferences for risk and ambiguity [27,35]. Participants made 40 choices between pairs of monetary outcomes, which allow us to parametrically and non-parametrically estimate their attitudes towards risk and ambiguity. The order in which the choice situations were presented was randomized separately for each participant. In each choice situation, the participant could select a certain payout of $5. The other option was a lottery with two possible outcomes: $0 or a positive dollar amount that varied from trial-to-trial. All possible lottery rewards ($5, $8, $20, $50, and $125) were fully crossed with all winning probability levels (13%, 25%, 38%, 50%, 75%) resulting in 25 unique *risky trials*. In these *risky* trials, both the reward and the probability of winning were precisely known. There were additional 15 trials in which the exact odds of winning were not known, which we call *ambiguous trials*. There were three possible levels of ambiguity (25%, 50%, 75%), each fully crossed with the same five possible rewards ($5, $8, $20, $50, and $125). Ambiguity was always centered on an equal chance of winning or not, which effectively replicated the classic Ellsberg design [36]. Fig 1 shows examples of screen shots from the exhibit.

Participants were instructed to respond truthfully. They were informed that each month one participant would be selected to receive payment based on one of her/his randomly selected choices. We note that these payment probabilities are quite low for the literature. But even though we could not pay every single participant as is usually done, our participants’ estimated risk attitudes were well within the range of the estimates obtained in previous laboratory studies that used more frequent incentives (e.g. [37]). Moreover, we note that our results replicate standard gender and wealth effects on risk attitudes and in addition replicate the laboratory findings on the effects of cloud coverage on risk taking [15]. We therefore conclude that our mechanism achieves stable performance similar to that achieved by other higher frequency of payment methods. Participants filled out a short questionnaire including questions about their age, gender, and relative wealth level (measured on a 5-point Likert scale) among others. The email addresses of the subjects were also collected and used to contact the winners. Their payment was send as gift cards via regular mail.

To investigate whether our participants’ behavior was affected by the weather we merged the behavioral data from our museum visitor participants with luminance measurements taken near the museum. The luminance data (surface radiation) was obtained from the U.S. Earth System Research Laboratory that collects luminance data in nine locations in the US. One of the stations is located in the vicinity of the museum in Sterling, VA near the Dulles airport. Data from this station can be downloaded free of charge at ftp://aftp.cmdl.noaa.gov/data/radiation/isis/ste/. Generally speaking, ‘luminance’ is a measurement of the amount of light that falls on the surface of the earth. Cloud cover, humidity, suspended particles in the atmosphere, time of day, time of year, and a number of other factors influence luminance. Several methods exist for measuring or estimating luminance [38]. The data we report here are derived from the US National Oceanographic and Atmospheric Administration’s Integrated Surface Irradiance Study (ISIS) Network and are provided in roughly 3-minute intervals. Measurements were made with a Total Solar Pyranometer, which measures broad field solar radiation flux density in Watts per square meter. More technical details about the measurement can be obtained from www.esrl.noaa.gov/gmd/grad/isis.

We note that a reader might be concerned that all subjects performed our risk assessment task under constant indoor illumination in the museum. This raises the possibility that some selection effect, or effect of time in the dimly lit museum might have shaped or contaminated our results. Solid physical and biological and evidence on the effects of light on brain function, however, mitigate this concern to some degree. First, we note that the light outside on a sunny day is typically 5 to 6 orders of magnitude greater in intensity than the light inside the museum and light intensity outside varies from day to day over about 3 orders of magnitude compared with the less than 1 order of magnitude variation inside the museum. Further, the biological and psychological effects of higher intensity outdoor luminance are now well-known to persist for hours or even for days. The standard light therapy for treating seasonal affective disorder, for example, is exposure to one hour of outdoor-intensity light each day. The effects of this single hour-long exposure produce measurable behavioral changes lasting for days [39]. Moreover, even minutes long exposure to outdoor light can significantly affect daily circadian rhythms [40]. In summary, it seems biologically unlikely that variation in exposure duration or intensity within the dim confines of the museum could account for our results. Nevertheless, it is important to interpret our results remembering that they relate to changes in the outdoor luminance level averaged over the hour(s) or day in which they participated in the study.

## Results and discussion

### Summary statistics on subjects and weather

**Fig 2:**
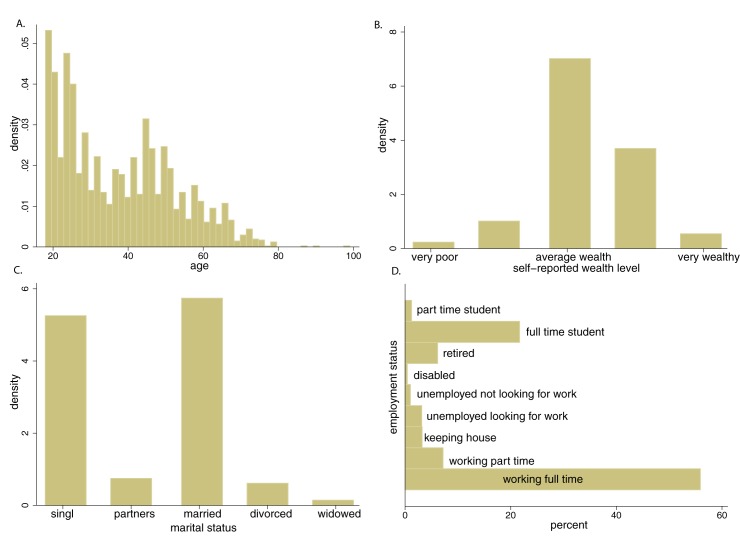
Characteristics of study participants. A: age, B: self-reported wealth level, C: marital status, D: employment status.

Included in this analysis are 2,530 (1,287 male, age: 37.4 mean +/-14.99 SD) participants. These participants gave informed consent, finished answering 40 questions and gave reasonable answers in the demographic questionnaire. Subjects who gave informed consent, but did not finish the full task are not included in this analysis. We did not exclude participants who completed the whole task but missed a small fraction of the questions due to an overly slow response. Overall, only 0.6% of the trials in our dataset are missing a decision. The maximum number of trials missed per subject was 6 out of 40, and the average was 0.24. Subjects who gave informed consent but reported being over 100 years old, or having more than 20 siblings were excluded from the analysis because we concluded that they did not take the task seriously. In total, 269 participants are excluded from the analysis because of the above reasons. Including them in the analysis does not change our luminance results. After accounting for the excluded participants, on average 7.13 people participated in the study each day (standard deviation: 4.39). The highest number of participants in a day was 21 and the lowest was 0. Fig 2 shows the distribution of ages, wealth, employment and marital status self-reported by the included participants. The participants seem to have understood and paid attention to the task. We conclude this from Table 2, where we present regression results that show that study participants selected the lottery more often as reward magnitude increased and as the probability of receiving the reward increased. Participants selected the lottery less often the more ambiguity it involved, consistent with generally observed patterns of ambiguity avoidance (for examples see [41]). We found higher levels of risk taking among male and wealthier participants as would be expected [42]. The decision-making patterns that we find in the museum visitors are thus consistent with a large body of previous experimental findings in the laboratory conditions.

**Table 2:** Understanding of the task. Logistic regression with a binary dependent variable *chose lottery* which is equal to 1 if the participant selected the lottery and 0 if the participant selected riskless option of $5. *reward* is the dollar amount associated with the lottery ($5, $8, $20, $50, or $125); *probability* is the probability of winning the *reward* (0.13, 0.25, 0.38, 0.5, or 0.75); *ambiguity* is the level of ambiguity associated with the lottery (.24, 0.5, or 0.74); *male* is a dummy variable equal to 1 if the participant is male; *wealth* is the self-reported wealth level ranging from 1 –very poor to 5 –very rich.

	*chose lottery*
*reward ($)*	0.004***
	(0.000)
*probability*	0.936***
	(0.009)
*ambiguity*	-0.098***
	(0.007)
*male*	0.042***
	(0.007)
*wealth*	0.016**
	(0.004)
*constant*	-0.210***
	(0.017)
N	100595

Robust standard errors clustered on participant.

* p<0.05

** p<0.01

*** p<0.001

**Fig 3:**
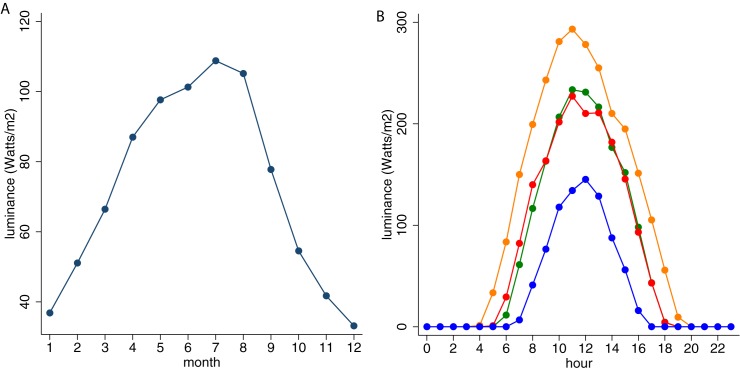
Observed luminance levels. A. Average monthly luminance measurements. B. Average hourly luminance levels in March (green), June (orange), September (red), and December (blue).

The geographical location of Washington, DC is well suited for studying the effects of luminance (and weather in general) on behavior. The area has highly variable weather conditions and is at an appropriate distance from the equator to experience varying seasonal levels of luminance. Fig 3A shows the average monthly (integrated) luminance levels throughout the year. These monthly differences reflect changes in the maximum daily luminance levels as well as the duration of positive luminance levels during each day. Fig 3B shows averaged hourly luminance levels in March (green), June (orange), September (red) and December (blue). As expected, luminance reaches highest (lowest) levels and above zero levels are present for the longest (shortest) part of the day in summer (winter) months. Daily levels of luminance are, of course, strongly affected by meteorological and environmental conditions such as cloud coverage, temperature, precipitation, and pollution. Therefore, luminance is not fixed for any day of the year, but rather varies substantially from day to day. The standard deviation in average daily luminance is equal to 33 Watt/m2 in the spring, 31 in the summer, 28 in the fall, and 18 in the winter. Table 3 includes summary statistics on all luminance and demographic variables that we use in the analysis.

**Table 3:** Summary statistics of independent variables.

	mean	st. dev.	min	max
*age*	37.4	14.99	18	99
*male*	0.51	0.5	0	1
*wealth*	3.26	0.75	1	5
*luminance (hour)*	166.8	114.46	0	617.92
*luminance (day)*	73.95	36.99	6.71	166.55
*luminance (last 2 days)*	150.62	70.15	26.45	322.51
*cloud coverage*	5.3	2.13	0	8

### Selection to participate in the study

One might reasonably worry that the participants in our study self-select into participating on certain days based on the luminance level, and that this self-selection may argue against drawing conclusions from these findings. To search for evidence of such selection effects we examined the relationship between luminance and: 1) number of participants, 2) participant age, 3) participant gender, and 4) participant self-reported wealth. We saw no evidence that any of these demographic properties of our subjects varied as a function of luminance. As shown in Tables 4–7, we determined this by regressing each of these properties against luminance level in that day and in the past two days. In summary, we did not find any relationship between the current and past luminance levels and participants’ individual characteristics (age, gender and wealth) known to affect preferences.

**Table 4:** Luminance does not affect the daily total number of volunteers who participate in the study. The table presents the results of OLS regression. *luminance (day)* is the daily luminance average; *luminance (last 2 days)* is the sum of the average luminance levels in the past two days.

*Dependent variable*: *total number of participants*			
*Luminance (day)*	-0.0030		-0.0076
	(0.0037)		(0.0052)
*luminance (last 2 days)*		0.0013	0.0039
		(0.0024)	(0.0033)
*constant*	4.7301***	4.3085***	4.4862***
	(0.3288)	(0.3919)	(0.3919)
N	561	561	561
R-squared	0.001	0.0007	0.0045

Robust standard errors in parentheses

+ p<0.01

* p<0.05

** p<0.01

*** p<0.001

**Table 5:** Luminance does not affect the age of volunteers who participate in the study. The table presents the results of OLS regression. *luminance (day)* is the daily luminance average; *luminance (last 2 days)* is the sum of the average luminance levels in the past two days.

*Dependent variable*: *age*			
*luminance (day)*	0.0037		0.0128
	(0.0080)		(0.0104)
*luminance (last 2 days)*		-0.0034	-0.0077
		(0.0042)	(0.0055)
*constant*	37.1204***	37.9109***	37.6015***
	(0.6571)	(0.6999)	(0.7387)
N	2528	2528	2528

Robust standard errors in parentheses

+ p<0.01

* p<0.05

** p<0.01

*** p<0.001

**Table 6:** Luminance does not affect the gender of volunteers who participate in the study. The table presents the results of logistic regression. *luminance (day)* is the daily luminance average; *luminance (last 2 days)* is the sum of the average luminance levels in the past two days.

*Dependent variable*: *male*			
*luminance (day)*	-0.0013		-0.0016
	(0.0011)		(0.0014)
*luminance (last 2 days)*		-0.0003	0.0003
		(0.0006)	(0.0007)
*constant*	0.1300	0.0747	0.1138
	(0.0890)	(0.0943)	(0.1001)
N	2528	2528	2528

Robust standard errors in parentheses

+ p<0.01

* p<0.05

** p<0.01

*** p<0.001

**Table 7:** Luminance does not affect the wealth of volunteers who participate in the study. The table presents the results of OLS regression. *luminance (day)* is the daily luminance average; *luminance (last 2 days)* is the sum of the average luminance levels in the past two days.

*Dependent variable*: *wealth*			
*luminance (day)*	0.0004		0.0008
	(0.0004)		(0.0005)
*luminance (last 2 days)*		-0.0001	-0.0004
		(0.0002)	(0.0003)
*constant*	3.2364***	3.2783***	3.2588***
	(0.0329)	(0.0358)	(0.0371)
N	2528	2528	2528

Robust standard errors in parentheses

+ p<0.01

* p<0.05

** p<0.01

*** p<0.001

Of course a failure to find such a correlation is not proof that no selection bias exists, but one might be encouraged by the fact that our findings replicate the results of an existing study. [15] conducted a laboratory study of the effects of cloud coverage on risk attitudes where selection-issues were absent as participants were randomly assigned to high and low cloud coverage treatments. Given that we replicate the results of this paper (see S1 and S2 Tables in supporting information), we take this to suggest that no major self-selection mechanism is likely to cloud our results.

While we cannot completely reject the idea that some form of self-selection operates in our study, we believe that it nevertheless makes an important contribution because of its scale. A study on this scale, conducted over such a long time period, is simply infeasible in the laboratory setting making our study a unique complement to the existing and future studies in this domain.

### Luminance results: Risk and ambiguity attitudes

**Fig 4:**
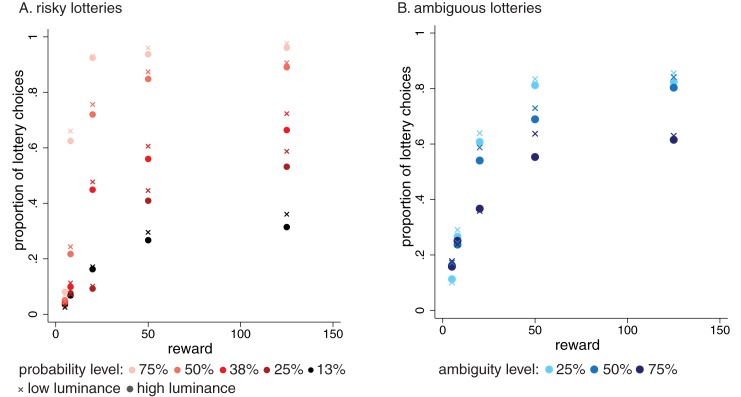
**Average proportion of lottery choices in the data at different levels of reward and probability (A) and different levels of reward and ambiguity (B).** Circles (crosses) correspond to choices made on days where the average luminance was above (below) the average of two past days.

Perhaps the simplest way to assess whether changes in luminance levels affect behavior under risk is to compare the average proportion of risky choices made when luminosity levels in the last two days have been above, versus below, the two-year luminance average. In Fig 4 we plot the average proportion of times that participants selected a lottery instead of the safe amount for different reward, probability and ambiguity levels when luminance levels increase or decrease. The circles (crosses) correspond to days that were preceded by two days with overall luminance levels above (below) the average. For a great majority of the lottery types, we see that crosses are above the circles indicating that people choose the lottery more often when exposed to lower levels of luminance. These differences are largest in choice situations when a representative participant would be indifferent between the lottery and a sure win of $5. The effects of luminance are smaller or nonexistent in choice situations where people have a clear preference for either a lottery or $5, for example when the lottery offers a small probability of a small reward or a large probability of a large reward.

Even though informative, this simple visual illustration of the data does not provide any information about significance, does not account for demographic factors that may be affecting the analysis, and does not allow us to infer anything about ambiguity attitudes which are confounded with risk attitude in this basic analysis. We address these problems using maximum likelihood techniques to estimate a structural model [43] that separates risk attitudes from ambiguity attitudes. In the model we use demographic variables known to affect decision-making under risk as covariates and explore whether these variables mediate the strength of the effect.

We assume a power utility function and incorporate ambiguity attitudes as in [44]. The expected utility from a lottery *(x*, *p*, *a)*, where *x* is the reward size, *p* is the probability of winning the reward, and *a* is the associated ambiguity level, is given by:

$$U(x,p,a)=(p+\beta \frac{a}{2})x^{\alpha }$$

where alpha is risk attitude and beta is attitude towards ambiguity to be estimated based on participants’ choices. Alpha smaller than (equal to, larger than) 1 indicates risk aversion (neutrality, seeking). Beta larger (smaller) than 0 indicates that the individual behaves as if the probability of winning was larger (smaller) than the objective probability of winning *p* (equal to 0.5 in our design) and therefore we classify the individual as ambiguity seeking (averse).

We model choice behavior (i.e. whether a person selected the risky lottery or the safe option) using a logistic choice function where the probability of choosing the risky lottery depends on the difference between the expected utilities of the risky (*EU*_*R*_) and safe option (*EU*_*S*_) as well as an independent and identically distributed (iid) error term with zero mean and variance parameter equal to sigma in the following way:

$$\mathrm{Pr}\left(ChoseRisky\right)=\frac{1}{1+exp(-\frac{{EU}_{R}-{EU}_{S}}{\sigma })}$$

where sigma is the structural noise parameter. We obtain reasonable parameter estimates (alpha = 0.455, beta = -0.365, sigma = 0.819, all significantly different from zero; maximum likelihood = -50796.662). In all of the analyses in this paper we cluster standard errors on the subject, to account for the fact that we have many observations coming from the same subject.

To estimate whether risk and ambiguity attitudes were significantly affected by weather, we replaced the risk and ambiguity parameters in our model with a linear combination of luminance level, demographic variables, and a constant and then estimated the model using maximum likelihood method. Table 8 shows the results of this analysis. (We ran the same analysis using the logarithm of luminance instead of luminance itself. These results, which are consistent but generally slightly weaker, are reported in S3 Table in the supporting information.) We found that people are significantly more risk averse when exposed to high luminance levels. This holds for each measure of luminance used: averaged hourly luminance at the hour when the participant completed the task, averaged daily luminance, and sum of averaged luminance levels in the past two days. This suggests that the total exposure to light affects individual risk taking. These effects remain significant when we control for the participants’ reported age, gender and wealth.

**Table 8:** Maximum likelihood estimates of risk and ambiguity attitude determinants. Each column shows the estimated coefficients and standard errors in parenthesis for different model specifications. *luminance (hour)* is the hourly average luminance level; *luminance (day)* is the daily luminance average; *luminance (last 2 days)* is the sum of the average luminance levels in the past two days. Higher ambiguity and risk estimates mean more risk and ambiguity tolerance.

	1	2	3	4	5	6	7
**risk attitude**							
*luminance (hour)*	-0.0001**			-0.0001+	-0.0001*		-0.0001*
	(0.0000)			(0.0000)	(0.0000)		(0.0000)
*luminance (day)*		-0.0003***		-0.0000	0.0000	-0.0002	
		(0.0001)		(0.0001)	(0.0001)	(0.0001)	
*luminance (last 2 days)*			-0.0002***	-0.0001+	-0.0001*	-0.0001*	-0.0001*
			(0.0000)	(0.0001)	(0.0001)	(0.0001)	(0.0000)
*age*					-0.0003	-0.0004	-0.0003
					(0.0002)	(0.0002)	(0.0002)
*male*					0.0511***	0.0508***	0.0511***
					(0.0062)	(0.0063)	(0.0063)
*wealth*					0.0203***	0.0202***	0.0203***
					(0.0046)	(0.0046)	(0.0046)
*constant*	0.4722***	0.4773***	0.4800***	0.4849***	0.4085***	0.4084***	0.4087***
	(0.0067)	(0.0079)	(0.0080)	(0.0086)	(0.0177)	(0.0179)	(0.0177)
**ambiguity attitude**							
*luminance (hour)*	0.0002			0.0001	0.0001		0.0002+
	(0.0001)			(0.0002)	(0.0002)		(0.0001)
*luminance (day)*		0.0004		0.0007	0.0008	0.0011*	
		(0.0004)		(0.0006)	(0.0006)	(0.0005)	
*luminance (last 2 days)*			-0.0001	-0.0005+	-0.0005+	-0.0005+	-0.0003
			(0.0002)	(0.0003)	(0.0003)	(0.0003)	(0.0002)
*age*					0.0012	0.0013	0.0012
					(0.0010)	(0.0010)	(0.0010)
*male*					0.0461	0.0462	0.0448
					(0.0283)	(0.0283)	(0.0283)
*wealth*					-0.0137	-0.0134	-0.0135
					(0.0196)	(0.0196)	(0.0196)
*constant*	-0.3931***	-0.3975***	-0.3462***	-0.3685***	-0.3991***	-0.4017***	-0.3890***
	(0.0238)	(0.0310)	(0.0341)	(0.0358)	(0.0783)	(0.0784)	(0.0777)
**noise (sigma)**							
*constant*	0.8184***	0.8183***	0.8184***	0.8180***	0.8144***	0.8145***	0.8145***
	(0.0154)	(0.0154)	(0.0155)	(0.0154)	(0.0153)	(0.0153)	(0.0153)
N	100515	100515	100515	100515	100515	100515	100515

Standard errors clustered on participant in parenthesis.

+ p<0.1

* p<0.05

** p<0.01

*** p<0.001

The effects of luminance on risk taking are not shockingly large, but are nevertheless quite substantial. Knowing that the standard deviation of the averaged luminance level over the past two days was equal to 46 (46, 26, 53) in the summer (fall, winter, spring) we can calculate that an increase in luminance from one standard deviation below the mean to one standard deviation above the mean would decrease risk taking (as measured with a power utility function) by 0.028 (6.154%) in summer and fall, 0.016 (3.517%) in the winter, and by 0.032 (7.033%) in the fall. This is a sizeable change, equivalent in our data to approximately half of the widely observed gender effect, equal to 0.051 in our sample (see Table 8). Thus a median man on a very sunny day and a median woman on a very cloudy day would show identical risk attitudes due simply to the weather.

The strength of the effect depends significantly on the reported age of the participant, with older people’s risk attitudes being more strongly affected by luminance levels (see Table 9). The coefficient on the luminance-age interaction term in model 2 in Table 9 is 0.0000054. This implies that if luminance in the past two days increased by one standard deviation, a 20-year-old would become more risk averse by 0.005, 0.005, 0.003 and 0.006 in the summer, fall, winter and spring respectively. The effect of a similar change in luminance for a 70-year-old would be much larger and equal to 0.017, 0.017, 0.0098 and 0.02 in summer, fall, winter and spring, respectively. The strength of the effect is not influenced by our measures of gender, or wealth.

**Table 9:** Maximum likelihood estimates of risk and ambiguity attitude determinants–age interaction. Each column shows the estimated coefficients and standard errors in parenthesis for different model specifications. *luminance (day)* is the daily luminance average; *luminance (last 2 days)* is the sum of the average luminance level yesterday and two days ago. Higher ambiguity and risk estimates mean more risk and ambiguity tolerance.

	1	2	3
**risk attitude (alpha)**			
*luminance (day)*	0.0001		0.0001
	(0.0002)		(0.0003)
*luminance (day) x age*	-0.0000+		-0.0000
	(0.0000)		(0.0000)
*luminance (last 2 days)*		0.0001	0.0001
		(0.0001)	(0.0001)
*luminance (last 2 days) x age*		-0.0000*	-0.0000
		(0.0000)	(0.0000)
*age*	0.0005	0.0007	0.0009
	(0.0005)	(0.0005)	(0.0006)
*male*	0.0501***	0.0506***	0.0504***
	(0.0063)	(0.0063)	(0.0063)
*wealth*	0.0199***	0.0192***	0.0195***
	(0.0046)	(0.0047)	(0.0047)
*constant*	0.3686***	0.3676***	0.3646***
	(0.0239)	(0.0235)	(0.0254)
**ambiguity attitude (beta)**			
*luminance (day)*	0.0005		0.0009
	(0.0011)		(0.0014)
*luminance (day) x age*	0.0000		0.0000
	(0.0000)		(0.0000)
*luminance (last 2 days)*		-0.0001	-0.0004
		(0.0006)	(0.0007)
*luminance (last 2 days) x age*		-0.0000	-0.0000
		(0.0000)	(0.0000)
*age*	0.0012	0.0013	0.0014
	(0.0023)	(0.0025)	(0.0026)
*male*	0.0449	0.0453	0.0461
	(0.0283)	(0.0283)	(0.0283)
*wealth*	-0.0122	-0.0124	-0.0133
	(0.0196)	(0.0197)	(0.0197)
*constant*	-0.4327***	-0.3830***	-0.4084***
	(0.1048)	(0.1109)	(0.1154)
**noise (sigma)**			
*constant*	0.8146***	0.8144***	0.8141***
	(0.0153)	(0.0153)	(0.0153)
N	100515	100515	100515

Standard errors clustered on participant in parenthesis.

+ p<0.1

* p<0.05

** p<0.01

*** p<0.001

While risk attitude is clearly influenced by the absolute current and past exposure to luminance, ambiguity is not. Neither the luminance on the hour and day of the experiment, nor the luminance levels in the past two days independently affect ambiguity attitude (Table 8, models 1–3). Instead ambiguity preferences seem to be influenced by changes in luminance levels. In the analysis presented in Table 8, model 6–7, where we control for the luminance level in the past two days we find that people are more ambiguity tolerant as the current luminance level increases (Table 8, model 6 and 7). In other words, the higher luminance today is relative to its levels in the last two days, the more optimistic about their chances of winning people are. In particular an increase in daily luminance by one standard deviation would result in individuals on average believing that their odds of winning are larger by 2 to 4% depending on the season. Alternatively, we can interpret the result as: keeping the current level of luminance fixed, participants are more ambiguity averse the higher was the luminance level over the past two days. We ran additional analysis, adding season dummy variables to model 4, presented in Table 8. The luminance effects remain the same with this control. Controlling for luminance, age and gender, participants are more risk averse in the fall and spring, relative to summer months.

### Luminance results: Dominance violations

**Fig 5:**
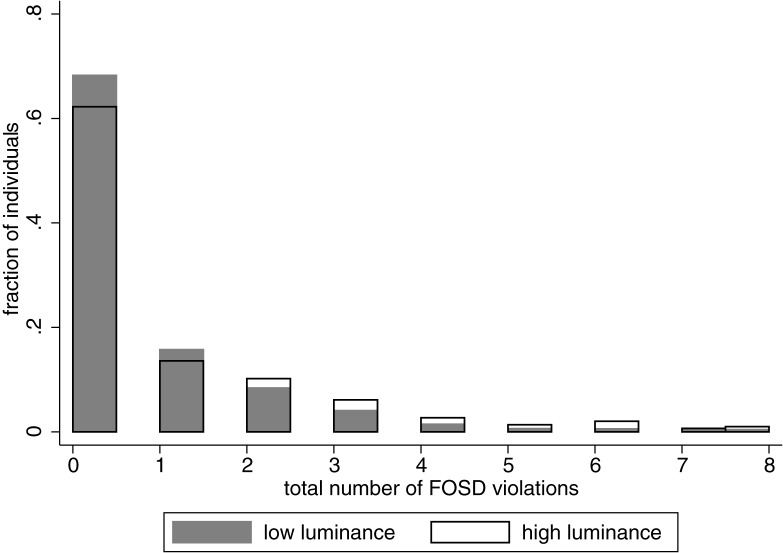
First order stochastic dominance (FOSD) violations in days with high luminance (white) and low luminance (gray). x-axis measures how many FOSD violations an individual committed. The height of the bars represents the fraction of the individuals in our sample who had that total level of FOSD violations. The average number of violations per individual is 0.674 (out of 8) with standard deviation equal to 1.297.

In our task each of the participants faced 8 questions in which they could violate first-order stochastic dominance (FOSD). These involved choosing between $5 for sure and a dominated lottery that would pay at most $5 with a probability strictly lower than 100%. An average participant violated FOSD in 0.674 out of 8 questions (standard deviation equal to 1.297), with some subjects not violating dominance at all and others violating it in all eight questions. To illustrate the distribution of dominance violations in our sample in different luminance conditions, in Fig 5 we show the proportion of our sample that violated dominance on low and high luminance days. Fig 5 suggests that dominance is violated more often when luminance is high.

To assess the significance of this finding, we used a logistic regression on the subset of choices that allowed for violations of the FOSD to assess whether the likelihood of choosing the dominated lottery is influenced by luminance. Table 10 shows that on days with higher luminance levels, participants were more likely to violate first-order stochastic dominance. Moreover the effect was strengthened if the luminance on the two preceding days was low suggesting that relative changes in luminance are important for rationality. Controlling for the current luminance level, the higher the average luminance in the last two days was, the more rational the behavior. Interestingly, the effect of luminance on dominance violations was much stronger for male participants (Table 11). While men in general violated dominance less than women, as the luminance level increases they started to violate dominance more (Table 11). Older people were more likely to violate dominance independent of the luminance level consistent with previous results [35].

**Table 10:** Logistic regression with a binary dependent variable equal to 1 if participant violated FOSD on a trial and 0 if not. Data includes only choices between $5 for sure and a $5 lottery. *luminance (hour)* is the hourly average luminance level; *luminance (day)* is the daily luminance average; *luminance (last 2 days)* is the sum of the average luminance level yesterday and two days ago.

	1	2	3	4	5	6	7
*luminance*	0.0008*			0.0001	0.0001		0.0009*
*hour*	(0.0004)			(0.0005)	(0.0005)		(0.0004)
*luminance*		0.0035**		0.0050**	0.0049**	0.0051***	
*day*		(0.0012)		(0.0018)	(0.0018)	(0.0015)	
*luminance*			0.0002	-0.0015*	-0.0015+	-0.0015*	-0.0004
*last 2 days*			(0.0006)	(0.0007)	(0.0008)	(0.0008)	(0.0006)
*probability*	1.6085***	1.6090***	1.6077***	1.6097***	1.6122***	1.6122***	1.6111***
	(0.1502)	(0.1502)	(0.1502)	(0.1502)	(0.1504)	(0.1504)	(0.1504)
*ambiguity*	1.8636***	1.8649***	1.8619***	1.8661***	1.8715***	1.8715***	1.8692***
	(0.0922)	(0.0921)	(0.0923)	(0.0921)	(0.0924)	(0.0924)	(0.0924)
*age*					0.0098**	0.0098**	0.0098**
					(0.0030)	(0.0030)	(0.0030)
*male*					-0.1848*	-0.1844*	-0.1923*
					(0.0880)	(0.0883)	(0.0880)
*wealth*					-0.0778	-0.0776	-0.0758
					(0.0669)	(0.0670)	(0.0671)
*constant*	-3.7606***	-3.8804***	-3.6502***	-3.7867***	-3.8156***	-3.8158***	-3.7511***
	(0.1193)	(0.1292)	(0.1354)	(0.1395)	(0.2450)	(0.2451)	(0.2453)
N	20224	20224	20224	20224	20224	20224	20224

Standard errors clustered on subject in parenthesis.

+ p<0.01

* p<0.05

** p<0.01

*** p<0.001

**Table 11:** Logistic regression with binary dependent variable equal to 1 if participant violated FOSD on a trial and 0 if not. Data includes only choices between $5 for sure and a $5 lottery. *luminance* is the hourly average luminance level in model 1; the daily average in model 2; and the sum of the average luminance level yesterday and two days ago in model 3.

	1—hour	2—day	3—past
*probability of winning*	1.6122***	1.6125***	1.6117***
	(0.1504)	(0.1504)	(0.1504)
*ambiguity level*	1.8716***	1.8723***	1.8705***
	(0.0923)	(0.0923)	(0.0925)
*luminance*	-0.0003	0.0010	-0.0041
	(0.0021)	(0.0062)	(0.0029)
*luminance x age*	-0.0000	0.0000	0.0001*
	(0.0000)	(0.0001)	(0.0000)
*luminance x male*	0.0021**	0.0055*	0.0025*
	(0.0008)	(0.0024)	(0.0012)
*luminance x wealth*	0.0001	-0.0006	-0.0001
	(0.0007)	(0.0021)	(0.0009)
*age*	0.0109*	0.0062	-0.0035
	(0.0054)	(0.0071)	(0.0069)
*male*	-0.5561***	-0.6142**	-0.5747**
	(0.1543)	(0.1946)	(0.1935)
*wealth*	-0.0947	-0.0258	-0.0539
	(0.1148)	(0.1447)	(0.1384)
*constant*	-3.6066***	-3.7372***	-3.0740***
	(0.3902)	(0.4705)	(0.4599)
N	20224	20224	20224

Standard errors clustered on subject.

+ p<0.01

* p<0.05

** p<0.01

*** p<0.001

### Luminance results: Choice consistency

To obtain a choice consistency measure, for each individual and for each reward (probability) level we calculated the number of times that the individual switched between choosing $5 for sure and a lottery as the probability (reward) level increased. We then summed up these numbers across all reward and probability levels to obtain our final measure of consistency in choice. This is similar to just counting how many times a person switched from the option on the left to the option on the right in a traditional ‘list-price’ experiment such as used in [37]. In our design, however, consistency is not made as obvious to the subject as each choice situation is presented independently on a separate screen. The only other difference here is that we also have to account for the fact that in our design not only probability levels, but also reward levels change. Of course, a consistent chooser would switch for each reward (probability) level at most once as the probability (reward) level increases. Overall, a completely noiseless chooser would switch between 0 and 6 times depending on his/her risk attitude. In the choice consistency analysis we include only risky trials where the odds of winning are known precisely as it is not clear what the switching pattern of a consistent chooser should be in the ambiguous trials.

**Fig 6:**
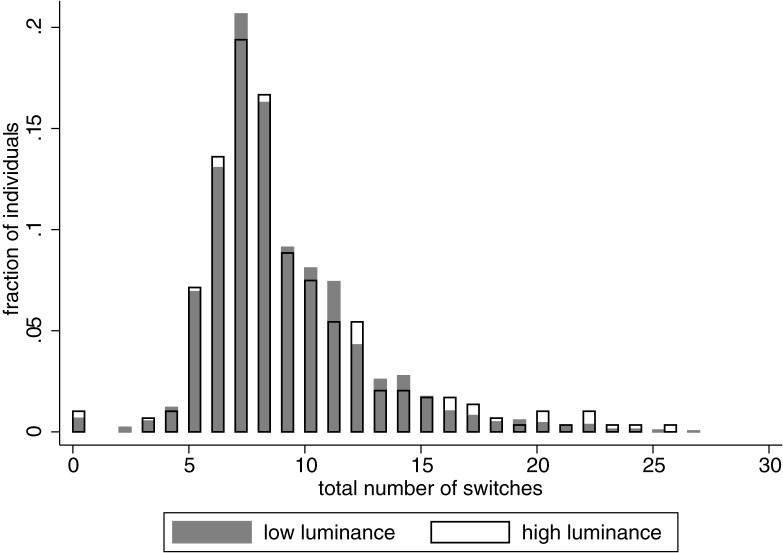
Choice consistency in days with high luminance (white) and low luminance (gray). A completely consistent chooser would switch between 0 and 6 times depending on preference. On average participants switched 8.75 times (standard deviation: 3.43).

On average people switched 8.75 times (standard deviation 3.43). Some of the participants did not switch at all and kept choosing only the lottery or $5 throughout the task. A subject who switched most frequently did it 27 times and this participant’s choices could be well described as apparently random. Fig 6 describes the distribution of switches in our population. As shown in Table 12, the absolute level of luminance did not affect how consistent were our subjects. However, controlling for the luminance in the last two days (on the day of participation), the higher the luminance level was on the day of the museum visit (in the last two days), the less (more) consistent our participants were. Neither age, gender nor wealth affected the strength of this effect.

**Table 12:** Inconsistency in choice. OLS regression with the number of times that the participant switched his choice as dependent variable. *luminance (hour)* is the hourly average luminance level; *luminance (day)* is the daily luminance average; *luminance (last 2 days)* is the sum of the average luminance level yesterday and two days ago.

	1	2	3	4	5	6	7
*luminance*	0.0005			-0.0006	-0.0006		0.0008
*hour*	(0.0006)			(0.0008)	(0.0008)		(0.0006)
*luminance*		0.0033+		0.0087**	0.0085**	0.0072**	
*day*		(0.0018)		(0.0030)	(0.0030)	(0.0024)	
*luminance*			-0.0010	-0.0035**	-0.0034**	-0.0034**	-0.0016
*last 2 days*			(0.0010)	(0.0012)	(0.0012)	(0.0012)	(0.0011)
*age*					0.0108*	0.0107*	0.0108*
					(0.0047)	(0.0047)	(0.0047)
*male*					-0.3107*	-0.3130*	-0.3246*
					(0.1363)	(0.1362)	(0.1364)
*wealth*					-0.0153	-0.0165	-0.0122
					(0.0939)	(0.0939)	(0.0940)
*constant*	8.6637***	8.5013***	8.9006***	8.7285***	8.5374***	8.5366***	8.6421***
	(0.1204)	(0.1522)	(0.1613)	(0.1713)	(0.3611)	(0.3611)	(0.3597)
N	2528	2528	2528	2528	2528	2528	2528

+ p<0.01

* p<0.05

** p<0.01

*** p<0.001

## Conclusions

It is now well-established that light exposure affects essentially all aspects of animal life and influences affective states in humans. In the most extreme cases, when light exposure is limited people become seasonally depressed–a mental state often associated anecdotally with altered risk preferences. And in fact, these biological effects of light are mediated through neurobiological pathways now known to be involved in preference regulation [45–47]. In this paper we tested the neurobiological and psychological hypothesis that either relative or absolute light levels (both of which are encoded neurobiologically) can influence our most basic preferences: risk attitude, ambiguity attitude, choice consistency and propensity to choose dominated options. We used an incentive-compatible task to estimate these preferences in a total of 2530 participants, over a period of two years. The study took place in the US National Academy of Sciences Museum in Washington, DC. This is an ideal geographical location for such a study due to a large seasonal and daily variation in luminance in this region.

Previous papers investigating the relationship between weather and economic decision-making have focused on cloud coverage [9,10,15] and seasonally and geographically varying duration of light during the day [11]. Part of the reason for this may be that precisely calibrated luminance measurements are available in only nine locations in the U.S. We also acknowledge that especially for experimental studies, it is usually not feasible to collect daily behavioral measurements, and therefore for recruitment, experimenters have to rely on substantial changes to the widely available forecasted weather variables. Although common sense suggests that cloud coverage and luminosity are closely related, this relationship is in fact quite complex and remarkably non-monotone. Cloud coverage is a relatively simple measurement approximating the percentage of sky covered by clouds but the precise structure of the cloud coverage can have quite complex effects on luminance, which is the biological variable of real interest [48]. The altitude of the clouds, their thickness, the under-cloud atmosphere, the incident angle and intensity of solar radiation and pollution are additional factors that determine the amount of light that passes through clouds. Under many circumstances increases in cloud cover can actually increase surface luminance, for example a surface fog which can often trap photons and lead to oddly bright conditions [24]. Overall, cloud coverage alone has little to do with the light exposure at the earth surface level, a point relevant to previous studies of this issue. In our dataset, CloudCoverage does explain some daily variation in luminance, but only 7% of that variation.

We chose to focus directly on luminance because of its known effects on animal behavior and affect in humans. A very simplistic description of the biological mechanism through which luminance affects decision-making under risk could be summarized as follows: After the light falls on the retina, it is then transmitted to the hypothalamus via a dedicated absolute light level sensor which is distinct from the sensors we employ for visual perception. In the hypothalamus these accurate measures of luminance influence daily and annual behavioral rhythms in preferences ranging from food choice to mate choice. These changes in preferences doubtless reflect the fact that the hypothalamus is responsible for regulating hormones and neurotransmitters that govern body functions ranging from thirst to hunger, sleep, body temperature mood, and even sex drive.

Importantly, there is every reason to believe that some of these effects are mediated through strong anatomical connections between the hypothalamus and brain regions known to be involved in decision-making under risk. In fact, functional connections between this area and the ventromedial prefrontal cortex and orbitofrontal cortex (areas critical for decision-making) have now been demonstrated in choice tasks and the level of activity in the hypothalamus projected to these areas has now been shown to regulate risk attitude (for reviews see [8,49,50]). Given this neurobiological interconnectivity, the demonstrated influence of these light sensitive neural systems on risk attitude, and the psychological demonstration that light levels influence mood which is known to influence risk attitude as well, there seems every reason to suspect that luminance level should directly influence preferences in significant ways.

Consistent with this idea, previous research has shown that food and water deprivation, which modulates both mood and hypothalamic activity, also affects individual risk preferences and not only for food [51] but also for monetary rewards [52,53]. In line with these findings, hungry shoppers have been shown to purchase more of non-food items than sated shoppers further suggesting that utility is generally, even for non-food items, affected by hunger’s impact on the hypothalamus [54]. Not only hunger and thirst but also circadian rhythms and sleep deprivation, other features that regulate hypothalamic activation, have been shown to affect behavior. [55] found that at times of the day mismatched with their circadian rhythms, people tend to take more risks. [56] find that sleep deprivation affects people’s willingness to take risks. There is now extensive evidence that limited light exposure (rather than cloud coverage, rainfall or atmospheric pressure) affects mood, even causing depression in some people [57]. Even stock markets are affected by day to night duration and mostly in countries far from the equator, where the variation in day length throughout the year is the largest and associated changes in mood most prevalent [11]. And establishing a truly causal link in the relationship between light and these behavioral features, artificial light therapy is now widely acknowledged to be the most effective remedy for seasonal depression, *or seasonal affective disorder*, and is known to operate via neural circuits in the hypothalamus [58]. With all this evidence available we hypothesized that light exposure would affect decision-making under risk. Even though we largely drew on the literature in neuroscience and psychology to form our hypothesis, our data does not allow us to verify that neurobiological connections between the eye and hypothalamus are the causal mechanism at work. Nevertheless the predictions that we built based on the existing evidence are all confirmed in the data.

Increased light exposure in the last two days, on the day of the experiment or at the hour around which the participant participated in the study all lead to more risk-aversion. Interestingly, the effect of luminance on risk taking was stronger for older participants which is in line with the evidence that older people are more vulnerable to weather and climate changes [59]. Only at a first sight are our findings contrary to an earlier study on the relationship between cloud coverage and risk-taking [15] which found that on cloudy days people take less risks. This is likely caused by the fact that there is not a monotone relationship between cloud coverage and luminance (see footnote 2). Nevertheless, to examine this issue we reran our analysis with cloud coverage as the explanatory variable to compare our data with this study. It is reassuring that we obtain qualitatively the same findings as [15] (see S1 and S2 Tables in the supporting information). On days with more cloud coverage people are more risk averse but only when current cloud coverage is very different from cloud coverage in the previous six days (S2 Table). In S2 Table, that finds the significant effect of cloud coverage on behavior, we followed [15] and included only data from days when the relative cloud coverage score (equal to current cloud coverage–average cloud coverage in the last 6 days) was in the top and bottom 10%. The absolute level of cloud coverage in our data does not explain variation in risk attitudes at all (S1 Table). This finding is similar to an earlier finding by [9] that stock market returns differ only for the most and least cloudy days and there is no effect at non-extreme levels of cloud coverage. Of course this implies that more research is needed to understand the mechanism through which different weather parameters affect decision-making.

We confirmed our hypothesis that relatively higher exposure to light leads to more optimistic beliefs and therefore more ambiguity tolerance. However, the absolute level of luminance did not affect ambiguity preferences, but instead relative changes in luminance did. The higher was current daily luminance level or/and the lower was luminance over the past two days, the more ambiguity tolerant people were. This is consistent with previous findings in the financial markets that analysts have more pessimistic beliefs about earnings in the fall [60] as well as with a study by [28] that negative (positive) mood increases (decreases) subjective probability of different death causes.

We note that in a recent working paper, [17] surveying a representative sample of Dutch citizens in a month of January, found that increased cloud coverage is associated with more ambiguity tolerance. The authors interpret departures from ambiguity aversion as a “mistake” and explain their result as subjects making wiser choices when in a sad mood. Whether ambiguity aversion (or risk aversion) is a behavioral mistake rather than individual’s trait is not the question that we address in this paper. Nevertheless consistent with the idea in [17] we found that light exposure affected people’s propensity to make rational decisions. Overall, participants were more inconsistent and more likely to violate dominance during increased light exposure, with the effects getting stronger the higher was current luminance relative to luminance in the past two days. This is in line with earlier findings that bad mood improves memory and ability to discriminate between different options [61] and that performance improves on analytical tasks under negative affect [34]. Increased luminance however did not make our participants more ambiguity averse–a point which may be policy relevant.

The observed effects are far from dramatic, which we find encouraging. While without doubt weather affects individual behavior at the same scale as does gender, in the end it does not fundamentally change how we behave and what we like. Nevertheless, when many market participants’ preferences shift in the same direction, this could create substantial market-level effects of luminance.

Our results contribute not only to the literature on weather, affect and decision-making but also to the long-standing discussion on ambiguity preferences in relation to risk preferences and rationality in choice. In particular, since we find that preferences for ambiguity and risk are differentially affected by light exposure, this suggests that the distinction between preference for known and unknown risks (first noted by [25]) may exist even at a biological level of analysis. In line with this finding, other research has previously shown that risk and ambiguity preferences are only weakly correlated [26,27]. Research on aging has yielded similar conclusions, demonstrating different lifespan patterns for attitudes towards risk and ambiguity [35,62–64].

Light exposure, in contrast to other weather variables such as cloud coverage or barometric pressure, is something that we can easily manipulate not only by spending more time outdoors but also with artificial methods like the use of specially designed lamps that imitate natural light indoors. Artificial light therapy is so successful in fighting depression, but one cannot help but wonder to what extent light therapy prescribed to depression sufferers affects their everyday decision-making. More importantly, we cannot help but note that manipulating the indoor luminance levels–the overhead light intensity–in markets like the New York Stock Exchange ought to have an effect on market volatility and risk premiums.

## Supporting information

S1 Fig The sequence of the experiment–consent, task, demographics. (EPS)

S1 Table Maximum likelihood estimates of risk and ambiguity attitude determinants. *cloud coverage (today)* measures the amount of cloud coverage in oktas from 0—clear sky to 8—overcast. *cloud coverage (last 2 days)* is the sum of cloud coverage measurements in the last two days. *wealth* is self-reported wealth measure, with values ranging from 1 (extremely poor) to 5 (extremely rich).
(DOCX)

S2 Table Maximum likelihood estimates of risk and ambiguity attitude determinants for the days with extremely high (top 10%) and extremely low (bottom 10%) cloud coverage relative to the average in the past six days. *cloud coverage* measures the amount of cloud coverage in oktas, from 0—clear sky to 8—overcast. *wealth* is self-reported wealth measure, with values ranging from 1 (extremely poor) to 5 (extremely rich).
(DOCX)

S3 Table Maximum likelihood estimates of risk and ambiguity attitude determinants. *log(luminance (hour))* is the logarithm of the hourly average luminance level; *log(luminance (day))* is the logarithm of the daily luminance average; *log(luminance (last 2 days))* is the log of the sum of the average luminance levels in the past two days. Higher ambiguity and risk estimates mean more risk and ambiguity tolerance.
(DOCX)

S1 Data (CSV)

S1 Task Instructions (DOCX)
