# Supplementary material for: Let the sunshine in? The effects of luminance on economic preferences, choice consistency and dominance violations
Source: PLoS One. 2017 Aug 4;12(8):e0181112. doi: 10.1371/journal.pone.0181112 (PMC5544238; doi:10.1371/journal.pone.0181112)
Supplement: S1 Table — cloud coverage (today) measures the amount of cloud coverage in oktas from 0—clear sky to 8—overcast. cloud coverage (last 2 days) is the sum of cloud coverage measurements in the last two days. wealth is self-reported wealth measure, with values ranging from 1 (extremely poor) to 5 (extremely rich). (DOCX) [file pone.0181112.s002.docx]

|  |  |  |  |  |
| --- | --- | --- | --- | --- |
|  | 1 | 2 | 3 | 4 |
| **risk attitude (alpha)** |  |  |  |  |
| *cloud coverage (today)* | -0.0014 |  | -0.0012 | -0.0012 |
|  | (0.0015) |  | (0.0015) | (0.0015) |
| *cloud coverage (last 2 days)* |  | -0.0006 | -0.0005 | -0.0006 |
|  |  | (0.0008) | (0.0009) | (0.0009) |
| *age* |  |  |  | -0.0004+ |
|  |  |  |  | (0.0002) |
| *male* |  |  |  | 0.0505*** |
|  |  |  |  | (0.0063) |
| *wealth* |  |  |  | 0.0207*** |
|  |  |  |  | (0.0046) |
| *constant* | 0.4627*** | 0.4623*** | 0.4672*** | 0.3910*** |
|  | (0.0088) | (0.0101) | (0.0119) | (0.0201) |
| **ambiguity attitude (beta)** |  |  |  |  |
| *cloud coverage (today)* | -0.0047 |  | -0.0044 | -0.0026 |
|  | (0.0065) |  | (0.0066) | (0.0067) |
| *cloud coverage (last 2 days)* |  | -0.0020 | -0.0015 | -0.0025 |
|  |  | (0.0041) | (0.0041) | (0.0042) |
| *age* |  |  |  | 0.0013 |
|  |  |  |  | (0.0010) |
| *male* |  |  |  | 0.0450 |
|  |  |  |  | (0.0282) |
| *wealth_num* |  |  |  | -0.0130 |
|  |  |  |  | (0.0196) |
| *constant* | -0.3398*** | -0.3432*** | -0.3256*** | -0.3569*** |
|  | (0.0370) | (0.0455) | (0.0530) | (0.0881) |
| **noise (sigma)** |  |  |  |  |
| *constant* | 0.8189*** | 0.8183*** | 0.8182*** | 0.8146*** |
|  | (0.0155) | (0.0154) | (0.0154) | (0.0153) |
| N | 100595 | 100555 | 100555 | 100555 |
| Standard errors clustered on participant in parenthesis. + p<0.1, * p<0.05, ** p<0.01, *** p<0.001 | | | | |
