# Supplementary material for: Let the sunshine in? The effects of luminance on economic preferences, choice consistency and dominance violations
Source: PLoS One. 2017 Aug 4;12(8):e0181112. doi: 10.1371/journal.pone.0181112 (PMC5544238; doi:10.1371/journal.pone.0181112)
Supplement: S2 Table — cloud coverage measures the amount of cloud coverage in oktas, from 0—clear sky to 8—overcast. wealth is self-reported wealth measure, with values ranging from 1 (extremely poor) to 5 (extremely rich). (DOCX) [file pone.0181112.s003.docx]

|  |  |  |
| --- | --- | --- |
|  | 1 | 2 |
| **risk attitude (alpha)** |  |  |
| *cloud coverage* | -0.0068** | -0.0072** |
|  | (0.0023) | (0.0023) |
| *age* |  | -0.0007 |
|  |  | (0.0005) |
| *male* |  | 0.0432** |
|  |  | (0.0145) |
| *wealth* |  | 0.0243* |
|  |  | (0.0104) |
| *constant* | 0.4824*** | 0.4103*** |
|  | (0.0141) | (0.0391) |
| **ambiguity attitude (beta)** | | |
| *cloud coverage* | 0.0012 | 0.0047 |
|  | (0.0112) | (0.0112) |
| *age* |  | 0.0022 |
|  |  | (0.0025) |
| *male* |  | 0.0231 |
|  |  | (0.0695) |
| *wealth* |  | -0.0208 |
|  |  | (0.0408) |
| *constant* | -0.3062*** | -0.3493* |
|  | (0.0586) | (0.1624) |
| **noise (sigma)** |  |  |
| *constant* | 0.8454*** | 0.8415*** |
|  | (0.0356) | (0.0351) |
| N | 21062 | 21062 |
| Standard errors clustered on participant in parenthesis (530 clusters). | | |
| + p<0.1, * p<0.05, ** p<0.01, *** p<0.001 | | |
