# Supplementary material for: Let the sunshine in? The effects of luminance on economic preferences, choice consistency and dominance violations
Source: PLoS One. 2017 Aug 4;12(8):e0181112. doi: 10.1371/journal.pone.0181112 (PMC5544238; doi:10.1371/journal.pone.0181112)
Supplement: S3 Table — log(luminance (hour)) is the logarithm of the hourly average luminance level; log(luminance (day)) is the logarithm of the daily luminance average; log(luminance (last 2 days)) is the log of the sum of the average luminance levels in the past two days. Higher ambiguity and risk estimates mean more risk and ambiguity tolerance. (DOCX) [file pone.0181112.s004.docx]

|  | 1 | 2 | 3 | 4 | 5 | 6 | 7 |
| --- | --- | --- | --- | --- | --- | --- | --- |
| **risk attitude** |  |  |  |  |  |  |  |
| *luminance* | -0.0041 |  |  | -0.0010 | -0.0010 |  | -0.0019 |
| *hour* | (0.0025) |  |  | (0.0031) | (0.0031) |  | (0.0028) |
| *luminance* |  | -0.0173** |  | -0.0079 | -0.0079 | -0.0085 |  |
| *day* |  | (0.0055) |  | (0.0078) | (0.0078) | (0.0070) |  |
| *luminance* |  |  | -0.0209*** | -0.0150* | -0.0150* | -0.0156* | -0.0196** |
| *last 2 days* |  |  | (0.0057) | (0.0072) | (0.0072) | (0.0072) | (0.0063) |
| *age* |  |  |  | -0.0004 | -0.0004 | -0.0004 | -0.0004 |
|  |  |  |  | (0.0002) | (0.0002) | (0.0002) | (0.0002) |
| *male* |  |  |  | 0.0511*** | 0.0511*** | 0.0507*** | 0.0511*** |
|  |  |  |  | (0.0063) | (0.0063) | (0.0063) | (0.0063) |
| *wealth* |  |  |  | 0.0199*** | 0.0199*** | 0.0200*** | 0.0198*** |
|  |  |  |  | (0.0046) | (0.0046) | (0.0046) | (0.0046) |
| *constant* | 0.4745*** | 0.5276*** | 0.5577*** | 0.4902*** | 0.4902*** | 0.4913*** | 0.4849*** |
|  | (0.0127) | (0.0234) | (0.0281) | (0.0336) | (0.0336) | (0.0337) | (0.0327) |
| **ambituity attitude** | |  |  |  |  |  |  |
| *luminance* | -0.0060 |  |  | -0.0179 | -0.0179 |  | -0.0105 |
| *hour* | (0.0121) |  |  | (0.0150) | (0.0150) |  | (0.0133) |
| *luminance* |  | 0.0189 |  | 0.0569+ | 0.0569+ | 0.0367 |  |
| *day* |  | (0.0239) |  | (0.0344) | (0.0344) | (0.0313) |  |
| *luminance* |  |  | -0.0003 | -0.0210 | -0.0210 | -0.0257 | 0.0121 |
| *last 2 days* |  |  | (0.0270) | (0.0358) | (0.0358) | (0.0358) | (0.0301) |
| *age* |  |  |  | 0.0013 | 0.0013 | 0.0013 | 0.0013 |
|  |  |  |  | (0.0010) | (0.0010) | (0.0010) | (0.0010) |
| *male* |  |  |  | 0.0438 | 0.0438 | 0.0452 | 0.0433 |
|  |  |  |  | (0.0283) | (0.0283) | (0.0283) | (0.0283) |
| *wealth* |  |  |  | -0.0122 | -0.0122 | -0.0131 | -0.0118 |
|  |  |  |  | (0.0197) | (0.0197) | (0.0196) | (0.0197) |
| *constant* | -0.3376*** | -0.4439*** | -0.3646** | -0.4494** | -0.4494** | -0.4266** | -0.4106** |
|  | (0.0594) | (0.1002) | (0.1328) | (0.1546) | (0.1546) | (0.1547) | (0.1529) |
| **noise** |  |  |  |  |  |  |  |
| *constant* | 0.8182*** | 0.8184*** | 0.8185*** | 0.8143*** | 0.8143*** | 0.8147*** | 0.8145*** |
|  | (0.0155) | (0.0154) | (0.0155) | (0.0153) | (0.0153) | (0.0153) | (0.0154) |
| N | 100118 | 100515 | 100515 | 100118 | 100118 | 100515 | 100118 |
| Standard errors clustered on participant in parenthesis. + p<0.1, * p<0.05, ** p<0.01, *** p<0.001 | | | | | | | |
