# Supplementary material for: Let the sunshine in? The effects of luminance on economic preferences, choice consistency and dominance violations
Source: PLoS One. 2017 Aug 4;12(8):e0181112. doi: 10.1371/journal.pone.0181112 (PMC5544238; doi:10.1371/journal.pone.0181112)
Supplement: S1 Task Instructions — (DOCX) [file pone.0181112.s006.docx]

| **Consent form** | |
| --- | --- |
| Visitors may opt out of the study at anytime during the activity. Visitors, who opt out of the study, will be able to start over and decline participation in the study.  Visitors will make choices between two different lotteries in which the odds of winning are either revealed or occluded. They will do 40 rounds of choices. Each round will last a maximum of 10 seconds. The total activity, with instructions and questionaires should take less than 12 minutes. After completing the 40 trials, visitors over 18 who gave their consent will be asked for additional demographic information. Entering the additional demographic information will be optional. Visitors will not be eligible to collect a reward unless they enter the additional demographic information and provde an e-mail address. For all users over 18 years-of-age, the activity will collect age and gender, trial data, any additional deemographic information provded, and the dollear amount of one of their lottery results. Of the visitors who provided e-mail addresses, the Koshland Science Museum will randomly select subjects to receive a gift card between $5 - 125 based on their choices. The dollar value will depend on a randomly selected trial and the visitor’s choices within that trial.  Visitors chosen to receive an award will be contacted with instructions on how to receive their award via e-mail. If a visitor is selected who based on his or her choices would receive $0, he or she will not be notified of the selection.  In order to participate in the study, you must agree to the following consent form by pressing the accept button.  By pressing decline, you give up a chance to win money but can still do the task. | |
| **Task instructions** | |
| 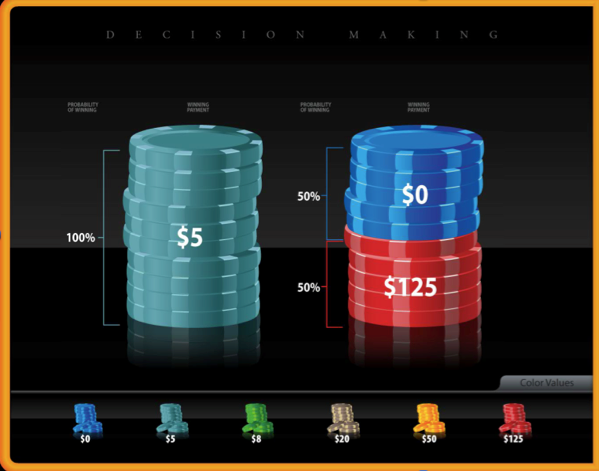 | In this game, you will be presented with a choice between two lotteries.  You will have 10 seconds to decide whether you prefer the lottery on the left or right. Press one of the poker chip stacks now to practice selecting a lottery.  Each lottery represents a stack of 100 colored poker chips. The size of the colored areas represents the number of chips of given color in the stack. The color represents the associated dollar amount: for example the navy blue poker chips represent $0; light blue $5 and red $125.  This means that if you were to pick the chip stack on the left, you would always get $5.  If you chose the stack on the right, you have equal chances of getting $125 . |
|  |  |
| 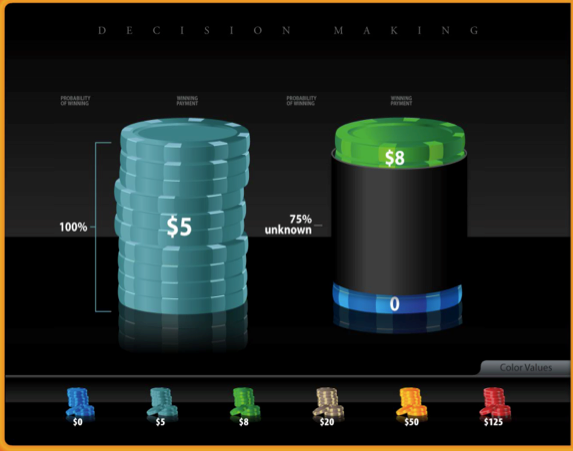 | In some trials, part of the chip stack will be hidden, so that you will only have part of the information about the number of chips. For example, the right lottery is 74% occluded. The actual probability of winning $8 could be anywhere between 13% to 87%.  Pay attention to your choices, one of them may count for payment.  Press start when you are ready! |
